# *Bacillus amyloliquefaciens, Bacillus velezensis*, and *Bacillus siamensis* Form an “Operational Group *B. amyloliquefaciens*” within the *B. subtilis* Species Complex

**DOI:** 10.3389/fmicb.2017.00022

**Published:** 2017-01-20

**Authors:** Ben Fan, Jochen Blom, Hans-Peter Klenk, Rainer Borriss

**Affiliations:** ^1^Co-Innovation Center for Sustainable Forestry in Southern China, College of Forestry, Nanjing Forestry UniversityNanjing, China; ^2^Bioinformatics and Systems Biology, Justus-Liebig-Universität GiessenGiessen, Germany; ^3^School of Biology, Newcastle UniversityNewcastle upon Tyne, UK; ^4^Fachgebiet Phytomedizin, Institut für Agrar- und Gartenbauwissenschaften, Humboldt Universität zu BerlinBerlin, Germany; ^5^Nord Reet UGGreifswald, Germany

**Keywords:** phylogenomics, *Bacillus subtilis* group, *Bacillus amyloliquefaciens*, *Bacillus taxonomy*, digital DNA–DNA hybridization, average nucleotide identity (ANI), average amino acid identity (AAI)

## Abstract

The plant growth promoting model bacterium FZB42^T^ was proposed as the type strain of *Bacillus amyloliquefaciens* subsp. *plantarum* (Borriss et al., 2011), but has been recently recognized as being synonymous to *Bacillus velezensis* due to phylogenomic analysis (Dunlap C. et al., 2016). However, until now, majority of publications consider plant-associated close relatives of FZB42 still as “*B. amyloliquefaciens*.” Here, we reinvestigated the taxonomic status of FZB42 and related strains in its context to the free-living soil bacterium DSM7^T^, the type strain of *B. amyloliquefaciens*. We identified 66 bacterial genomes from the NCBI data bank with high similarity to DSM7^T^. Dendrograms based on complete *rpoB* nucleotide sequences and on core genome sequences, respectively, clustered into a clade consisting of three tightly linked branches: (1) *B. amyloliquefaciens*, (2) *Bacillus siamensis*, and (3) a conspecific group containing the type strains of *B. velezensis, Bacillus methylotrophicus*, and *B. amyloliquefaciens* subsp. *plantarum*. The three monophyletic clades shared a common mutation rate of 0.01 substitutions per nucleotide position, but were distantly related to *Bacillus subtilis* (0.1 substitutions per nucleotide position). The tight relatedness of the three clusters was corroborated by TETRA, dDDH, ANI, and AAI analysis of the core genomes, but dDDH and ANI values were found slightly below species level thresholds when *B. amyloliquefaciens* DSM7^T^ genome sequence was used as query sequence. Due to these results, we propose that the *B. amyloliquefaciens* clade should be considered as a taxonomic unit above of species level, designated here as “operational group *B. amyloliquefaciens*” consisting of the soil borne *B. amyloliquefaciens*, and plant associated *B. siamensis* and *B. velezensis*, whose members are closely related and allow identifying changes on the genomic level due to developing the plant-associated life-style.

## Introduction

At the time of writing, the genus *Bacillus* (Gordon et al., 1973), consisted of 318 species with validly published names (http://www.bacterio.net/bacillus.html) with *Bacillus subtilis* as the type species (Cohn, 1872; Skerman et al., 1980). The industrial important species *B. subtilis, Bacillus amyloliquefaciens, Bacillus licheniformis*, and *Bacillus pumilus* are representing a group of phylogenetically and phenetically homogeneous species called, in the vernacular, the *B. subtilis* species complex (Fritze, 2004). For many years, it has been recognized that these species are hardly to distinguish on the basis of traditional phenotypic methods. Moreover, phylogenetic analysis of the 16S rRNA gene also fails to differentiate species within the complex due to the highly conserved nature of the gene (Rooney et al., 2009).

All members of this species complex are placed in 16S rRNA/DNA group 1. Its separation was based mainly on the significantly low DNA relatedness values experimentally determined by DDH, and their different fatty acid profiles (Priest et al., 1987). Besides the “original members” *B. subtilis, B. licheniformis*, and *B. pumilus*, early described by Gordon et al. (1973), many novel species belonging to the *B. subtilis* species complex have been described in last decades: *B amyloliquefaciens* (Priest et al., 1987), *Bacillus atrophaeus* (Nakamura, 1989), *Bacillus mojavensis* (Roberts et al., 1994), *Bacillus vallismortis* (Roberts et al., 1996), *Bacillus sonorensis* (Palmisano et al., 2001), *Bacillus velezensis* (Ruiz-García et al., 2005a), *Bacillus axarquiensis* (Ruiz-García et al., 2005b), *Bacillus tequilensis* (Gatson et al., 2006), *Bacillus aerius, Bacillus aerophilus, Bacillus stratosphericus, Bacillus altitudinis* (Shivaji et al., 2006), *Bacillus safensis* (Satomi et al., 2006), *Bacillus methylotrophicus* (Madhaiyan et al., 2010), *Bacillus siamensis* (Sumpavapol et al., 2010), *Bacillus xiamenensis* (Lai et al., 2014), *Bacillus vanillea* (Chen et al., 2014), *Bacillus paralicheniformis* (Dunlap C. et al., 2015), *Bacillus glycinifermentas* (Kim et al., 2015), *Bacillus oryzicola* (Chung et al., 2015), *Bacillus gobiensis* (Liu et al., 2016), and *Bacillus nakamurai* (Dunlap C. A. et al., 2016). *B. vanillea, B. oryzicola*, and *B. methylotrophicus* could not be corroborated as valid species and were identified as later heterotypic synonyms of either *B. siamensis* (Dunlap, 2015), or *B. velezensis* (Dunlap C. et al., 2016). *B. subtilis* has been subdivided into the three subspecies: *B*. *subtilis* subsp. *subtilis, B. subtilis* subsp. *spizizenii* (Nakamura et al., 1999), and *B. subtilis* subsp. *inaquosorum* (Rooney et al., 2009). In recent time, methods based on genome sequences (complete and WGS), such as ANI (Richter and Rosselló-Móra, 2009), AAI (Konstantinidis and Tiedje, 2005), dDDH (Meier-Kolthoff et al., 2013), and TETRA (Teeling et al., 2004), were used to finally discriminate a wide spectrum of bacterial taxons including the *B. subtilis* species complex (Federhen, 2015).

Some representatives of *B. amyloliquefaciens* were found plant-root-associated and to act beneficial on plant growth (Idriss et al., 2002). Reva et al. (2004) reported that seven *Bacillus* isolates from plants or soil are closely related to but distinct from *B. amyloliquefaciens* type strain DSM7^T^. These strains are more proficient for rhizosphere colonization than other members of the *B. subtilis* group (Hossain et al., 2015). *B. amyloliquefaciens* strains GB03 (Choi et al., 2014), and FZB42 (Chen et al., 2007) are widely used in different commercial formulations to promote plant growth.

With the advent of comparative genomics and the availability of an increasing number of whole genome sequences, it became possible to distinguish two subspecies within *B. amyloliquefaciens*: *B*. *amyloliquefaciens* subsp. *amyloliquefaciens* (type strain DSM7^T^), and *B. amyloliquefaciens* subsp. *plantarum* (type strain: FZB42^T^). Spectroscopic DDH performed with hydroxylapatite-purified chromosomal DNA from DSM7^T^ and FZB42^T^ yielded DNA-DNA relatedness values ranging between 63.7 and 71.2% which apparently did not sufficiently support discrimination of both taxons on the species level (Borriss et al., 2011). According to this view the subspecies “*plantarum*” represented a distinct ecotype of plant-associated *B. amyloliquefaciens* strains (Reva et al., 2004), which is increasingly used as biofertilizer and biocontrol agents in agriculture (Borriss, 2011).

Whilst many researchers are still using this classification (e.g., Hossain et al., 2015), recent phylogenomic studies showed a high degree of similarity between the genomes of the *B. methylotrophicus, B. velezensis, B. oryzicola*, and *B. vanillea* type strains, and the genome of the *B. amyloliquefaciens* subsp. *plantarum* type strain FZB42^T^ (= DSM 23117^T^ = BGSC 10A6^T^). Due to this finding it was proposed that the taxon *B. amyloliquefaciens* subsp. *plantarum* should be considered as a later heterotypic synonym of either *B. methylotrophicus* (Dunlap C. A. et al., 2015) or, more correctly due to priority rule, of *B. velezensis* (Dunlap C. et al., 2016). In spite of this increasingly complex taxonomic situation, we conducted here an extended phylogenomic analysis based on 66 core genomes displaying a high degree of similarity with the type strain of *B. amyloliquefaciens* DSM7^T^. It ruled out that three tightly linked clades including a conspecific group consisting of FZB42^T^, *B. methylotrophicus* KACC 13103^T^, and *B. velezensis* KCTC13012^T^, could be distinguished. The tight relatedness of the three clades consisting of representatives of *B. amyloliquefaciens, B. velezensis*, and *B. siamensis* was validated by *rpoB* gene sequence homology, and, ANI, AAI, dDDH, and TETRA analysis of the core genomes. We propose to introduce the term “operational group *B. amyloliquefaciens*” to underline their close phylogenomic relationship.

## Materials and methods

### Retrieval of *rpoB* sequences

Complete *rpoB* gene sequences with homology to *B. amyloliquefaciens* DSM7^T^ were retrieved from the respective genomes of *Bacillus* strains available at NCBI (http://www.ncbi.nlm.nih.gov/sutils/genom_table.cgi?organism$=$microb). Sequence comparisons were obtained by NCBI BlastN (http://blast.ncbi.nlm.nih.gov/Blast.cgi?CMD$=$Web&PAGE_TYPE$=$BlastHome).

### Alignment of DNA *rpoB* sequences

Alignment of DNA *rpoB* sequences was performed by the Clustal Omega program accessible at http://www.ebi.ac.uk/Tools/msa/clustalo/. A distance matrix was calculated from this alignment by DNA distance matrix calcuation (DNADIST program), and the matrix was then transformed into a tree by the NEIGHBOR program. In order to verify the accuracy of the tree multiple data sets were generated with the SEQBOOT program using 200 bootstrap replicates. A tree was built from each replicate with the DNADIST program, and then bootstrap values were computed with the CONSENSE program. The phylogenetic tree was visualized with TreeViewX (http://taxonomy.zoology.gla.ac.uk/rod/treeview.html). The programs used to construct the phylogenetic tree were obtained from the PHYLIP package, v.3.65 (Felsenstein, 1989), which is accessible at http://evolution.genetics.washington.edu/phylip.html.

### Comparative genome analysis

Comparative genome analysis was performed using the EDGAR 1.3 software framework. For orthology estimation EDGAR uses a generic orthology threshold calculated from the similarity statistics of the compared genomes (Blom et al., 2016; http://edgar.computational.bio.uni-giessen.de). A private project was constructed comprising 66 genomes closely related to *B. amyloliquefaciens* DSM7^T^ and selected other representatives of the *B. subtilis* species complex. To construct a phylogenetic tree for this project, around 2000 core genes were computed by pairwise iterative comparison of a set of genomes (Blom et al., 2016). In a following step multiple alignments of the core genes were generated using MUSCLE, non-matching parts of the alignment were masked by GBLOCKS and subsequently removed. The remaining parts of all alignments were concatenated to one large alignment. The PHYLIP package was used to generate a phylogenetic tree of this alignment, represented in newick format.

The EDGAR software framework was also used to calculate average nucleotide identity (ANI) and average amino acid identity (AAI), matrices for a selected set of genomes. The blast hits between the orthologous genes of the core of the selected genome were analyzed for their mean/median percent identity values. The recommended species cut-off was 95% for the ANI and AAI indices (Richter and Rosselló-Móra, 2009). In addition, JSpeciesWS (http://jspecies.ribohost.com/jspeciesws/) was used to determine ANIb (average nucleotide identity based on BLAST+) and ANIm (average nucleotide identity based on MUMmer) values by pairwise genome comparisons. Correlation indexes of their Tetra-nucleotide signatures (TETRA) were determined by using the JSpeciesWS software (Richter et al., 2016).

### Digital DNA–DNA hybridization (dDDH)

The genome-to-genome-distance calculator (GGDC) version 2.1 provided by DSMZ (http://ggdc.dsmz.de/) was used for genome-based species delineation (Meier-Kolthoff et al., 2013) and genome-based subspecies delineation (Meier-Kolthoff et al., 2014). Distances were calculated by (i) comparing two genomes using the chosen program to obtain HSPs/MUMs and (ii) inferring distances from the set of HSPs/MUMs using three distinct formulas. Next, the distances were transformed to values analogous to DDH. The DDH estimates were based on an empirical reference dataset comprising real DDH values and genome sequences. The DDH estimate resulted from a generalized linear model (GLM) which also provided the estimate's confidence interval (after the ± sign). Three formulas are available for the calculation: Formula: 1 (HSP length/total length), formula: 2 (identities/HSP length) and formula 3 (identities/total length). Formula 2, which is especially appropriate to analyze draft genomes, was used.

## Results

### Phylogenomics of the *B. subtilis* species complex

**Figure 1:**
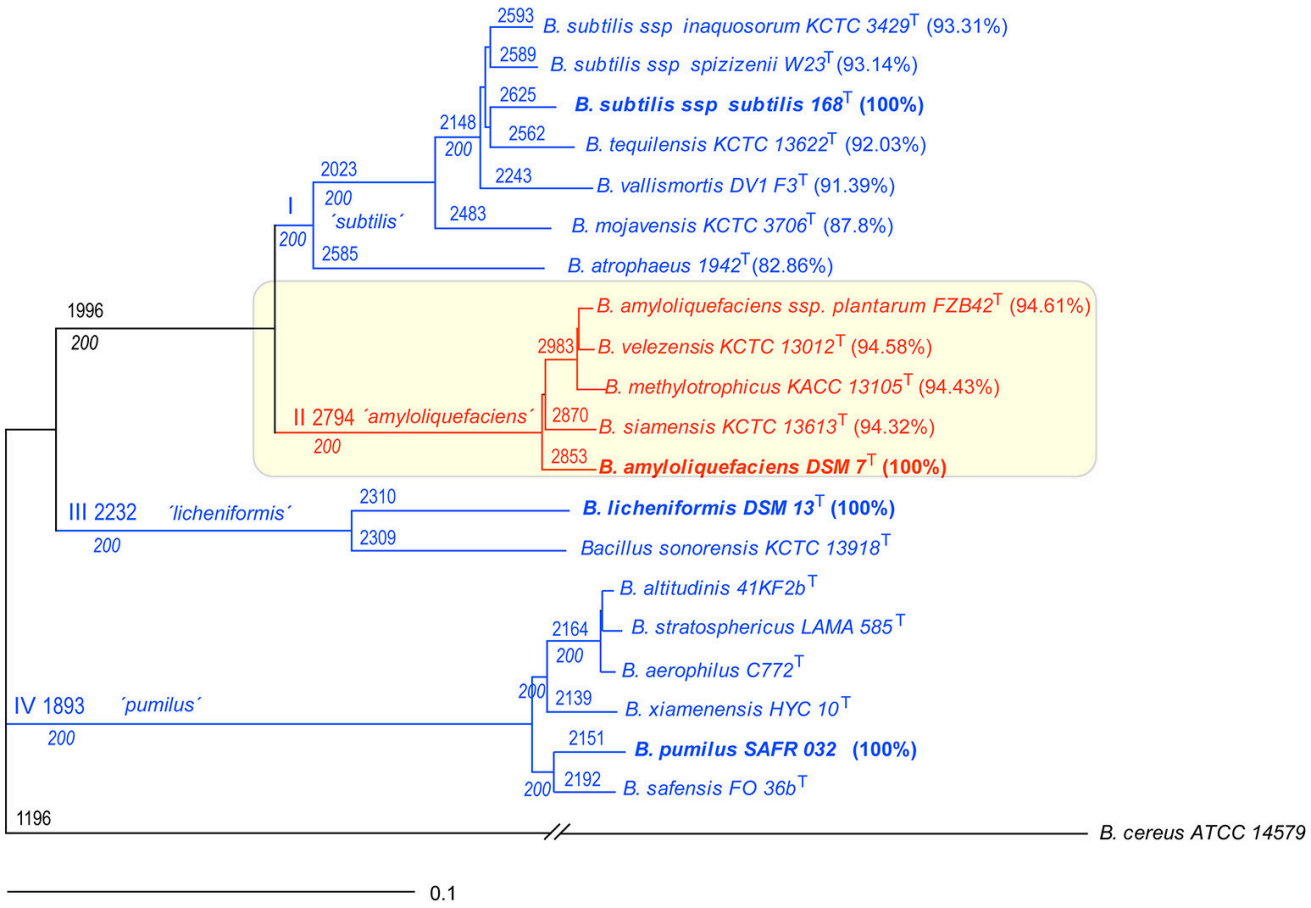
**Phylogeny of the *Bacillus subtilis* species complex based on the core genomes of representative type strains**. The core genome of *Bacillus cereus* ATCC14579 was used as outgroup. The roman letters at the branching points designate the four clades identified in this analysis. The numbers at the branching points designate the number of CDS calculated for the core genome of a given subset of genomes. Bootstrap values of 200 (100%) are indicated below the CDS numbers (see Materials and Methods). Percentage of identity according to type strains *B. subtilis* subsp. *subtilis* 168^T^, *B. amyloliquefaciens* DSM7^T^, *B. licheniformis* DSM13^T^, and *B. pumilus* SAFR032, respectively. Note that within clade II (“*amyloliquefaciens*”) the group with *B. amyloliquefaciens* subsp. *plantarum* FZB42^T^, *B. velezensis* KCTC 13012^T^, and *B. methylotrophicus* KACC 13105^T^ is conspecific. The same is true for the group within clade IV *(“pumilus”*) consisting of *B. altitudinis* 41KF2b^T^, *B. stratosphericus* LAMA 585^T^, and *B. aerophilus* C772^T^. The scale bar corresponds to 0.1 substitutions per site.

The core genomes of 20 type strains of the *B. subtilis* species complex were used for phylogenomic analysis applying the EDGAR software package (Figure 1). Four main monophyletic groups were corroborated by 100% bootstrap values. Clade I (*“subtilis”)* is early diverged into two branches comprising *B. atrophaeus*, and *B. subtilis* and its close relatives; clade II (“*amyloliquefaciens*”) comprises *B. amyloliquefaciens, B. siamensis*, and a conspecific group containing the type strains of *B. amyloliquefaciens* subsp. *plantarum, B. velezensis*, and *B. methylotrophicus*; clade III (“*licheniformis*”) consists of *B. licheniformis* and *B. sonorensis*; and clade IV (“*pumilus*”) comprises *B. pumilus, B. safensis, B. xiamenensis*, and a conspecific group involving the type strains of *B. altitudinis, B. stratosphericus*, and *B. aerophilus*. The members of clade II appeared closely related. This is indicated by the high number of orthologous CDSs (2794) shared by the five type strains of clade II. A similar cladogram has been published recently (Dunlap C. et al., 2016) suggesting that the *B. subtilis* species complex can be divided into four groups above species level, which need further characterization. We have directed our further analysis to clade II (named from now on “operational group *B. amyloliquefaciens*”), which clearly shows the highest degree of compactness.

### Phylogenetic analysis of clade II based on complete *rpoB* nucleotide sequence

It is obvious, that 16S rRNA sequences are not sufficient to discriminate representatives of the *B. subtilis* species complex. For example, comparison of the complete 16S rRNA sequences of *B. amyloliquefaciens* DSM7^T^ and *B. subtilis* 168^T^ revealed 99.48% identity (Table 1), which is well above of the recommended threshold of >98.65% for species delineation (Kim et al., 2014). In order to elucidate more precisely the phylogenetic and taxonomic relationship of the members of the *B. subtilis* species complex belonging to the “operational group *B. amyloliquefaciens,”* we used two methods. (i) Tetra correlation search (TCS, Richter et al., 2016) was performed with the complete genome of DSM7^T^ and (ii) the complete RNA polymerase beta-subunit (rpoB) gene of DSM7^T^ was used for BLASTN comparison with the corresponding sequences extracted from complete genomes or genome assemblies. Fifty-Two genomes, which were in range with the intraspecific Tetra-nucleotide signature correlation index (>0.99) were detected in the JSpecies data bank. The TCS value determined for *B. subtilis* was only 0.954, suggesting that using this alignment-free parameter allows discriminating of *B. subtilis* and *B. amyloliquefaciens* (Table 1). Complete rpoB gene sequencing has been proposed as phylogenetic marker (Klenk et al., 1994) and as a supplement to DDH (Adékambi et al., 2008). The power and potential of complete rpoB gene sequence in taxonomic, phylogenetic and evolutionary studies has been previously reported (Sharma and Patil, 2011). Our BLASTN search revealed that at least 66 genomes present in the NCBI data bank contain *rpoB* gene sequences with more than 98% identity to the *rpoB* gene from DSM7^T^, the type strain of *B. amyloliquefaciens* (Priest et al., 1987). For comparison, the *rpoB* gene from *B. subtilis* subsp. *subtilis* 168^T^ displayed only 90.3% identity to *B. amyloliquefaciens*. The rpoB gene identities among strains assigned as being *B. amyloliquefaciens, B. siamensis, B. amyloliquefaciens* subsp. *plantarum, B. methylotrophicus, B. velezensis*, and *B. vanillea* are listed in Table 1. The list of strains containing *rpoB* genes with high similarity to *B. amyloliquefaciens* DSM7^T^ includes also strains obviously not correctly assigned, such as *B. subtilis, Bacillus* sp., or *Paenibacillus polymyxa*. It is interesting to note that majority of the strains representing the conspecific *B. velezensis/B.methylotrophicus/B.amyloliquefaciens* subsp. *plantarum* group were isolated from plant sources, whilst *B. amyloliquefaciens sensu stricto* seems to be soil-borne. The main source of the salt tolerant *B. siamensis/B.vanillea* group was fermented plant food (Table 1).

**Table 1:** **Genomes containing *rpoB* sequences displaying ≥98% similarity to *B. amyloliquefaciens* DSM7^T^**.

**Strain**	**Accession**	**rpoB (%)**	**TETRA**	**ANIb**	**AAI**	**dDDH %**	**G+C**	**16S rRNA**	**Source**
***B. amyloliquefaciens***									
DSM7^T^	FN597644.1	**100**	**1.000**	**100**	**100**	**100** ± **0.0**	**46.1**	**100**	Soil, fermentation plant
LL3	CP002634.1	**100**	**0.99929**	**99.47**	**99.75**	**96.4** ± **1.12**	**45.7**	**99.87**	Fermented food (Korean bibimbap)
TA208	CP002627.1	**100**	**0.99945**	**99.28**	**99.65**	**95.2** ± **1.36**	**45.8**	**99.87**	Lab stock, overproducing guanosine
ATCC 13952	CP009748.1	**100**	**0.9995**	**99.26**	**99.64**	**95.4** ± **1.32**	**45.8**	**99.87**	Unknown
XH7	NC_017191.1	**100**	**0.9942**	**99.31**	**99.66**	**95.4** ± **1.33**	**45.8**	**99.87**	Unknown
CMW1	BBLH01000000	**99.50**	**0.99884**	**97.79**	**99.04**	**84.7** ± **2.56**	**46.0**	n.d	Japanese fermented soybean paste
***B. siamensis/B. vanillea***									
XY18^T^	gb|LAGT01000040.1|	**98.44**	**0.99702**	93.36	**97.82**	55.0 ± 2.72	46.3	**99.78**	Cured vanilla beans
JJC33M	JTJG01000000	**98.49**	**0.99678**	93.19	**97.78**	54.3 ± 2.71	45.7	n.d	Salted Thai crab product
KCTC 13613^T^	GCA_000262045.1	**98.30**	**0.99765**	93.27	**97.83**	54.7 ± 2.71	46.3	**99.69**	Sugar cane, Papaloapan, Mexico
***B. velezensis/B.methylotrophicus/B. amyloliquefaciens*** **subsp**. ***plantarum***									
W2	JOKF01000000	**98.50**	**0.99766**	93.45	**97.83**	55.8 ± 2.73	46.5	**99.61**	Saffron (*Crocus sativus*)
GR4-5	JYGH01000000	**98.49**	**0.99754**	93.14	**97.78**	55.0 ± 2.72	46.2	**99.48**	Korean ginseng rhizosphere
UCMB5033	emb|HG328253.1|	**98.49**	**0.99774**	93.41	**97.78**	56.3 ± 2.74	46.2	**99.68**	Cotton rhizosphere
Bs-916	gb|CP009611.1|	**98.49**	**0.9975**	93.38	**97.84**	56.2 ± 2.74	46.4	**99.67**	Paddy soil (rice)
JS25R	gb|CP009679.1|	**98.49**	**0.99782**	93.39	**97.78**	56.1 ± 2.74	46.4	**99.74**	Spikelets of wheat heads
SPZ1	AQGM00000000	**98.49**	**0.9976**	93.24	**97.77**	55.6 ± 2.73	46.2	**99.69**	Tributyrin enriched medium
ATCC12321	ARYD01000000	**98.49**	**0.99758**	93.22	**97.19**	55.6 ± 2.73	46.0	**99.69**	Spoiled starch
Bs006	LJAU01000000	**98.49**	**0.99698**	93.20	**97.81**	55.6 ± 2.73	45.8	n.d.	Banana roots, magdalena, colombia
916	AFSU00000000	**98.49**	**0.99697**	93.28	**97.81**	55.7 ± 2.73	46.4	n.d	Soil antagonist of rhizoctonia
B26	NZ_LGAT00000000	**98.49**	**0.99678**	93.47	**97.79**	55.9 ± 2.74	46.6	n.d	Switchgrass (*Panicum virgatum* l.)
OB9	LGAU00000000	**98.49**	**0.99628**	93.38	**97.79**	55.6 ± 2.73	46.7	n.d	Crude oil
NAU-B3	emb|HG514499.1|	**98.46**	**0.99744**	93.40	**97.21**	56.1 ± 2.74	45.9	**99.81**	Wheat rhizosphere
TrigoCor1448	gb|CP007244.1|	**98.46**	**0.9976**	93.48	**97.84**	55.7 ± 2.73	46.5	**99.67**	Wheat rhizosphere
EGD-AQ14	AVQH01000000	**98.46**	**0.99688**	93.19	**97.84**	55.7 ± 2.73	45.7	**99.67**	Saline desert plant rhizosphere
XK-4-1	LJDI00000000	**98.46**	**0.99754**	93.38	**97.84**	55.4 ± 2.73	46.0	n.d	Epiphyte cotton (*Gossypium* spp.)
629	NZ_LGYP00000000.1	**98.46**	**0.99754**	93.33	**97.79**	55.7 ± 2.73	46.5	n.d	Endophyte theobroma cacao
UNC69MF	JQKM01000000	**98.46**	**0.9966**	93.44	**97.80**	55.8 ± 2.73	46.5	n.d	Not reported
FZB42^T^	gb|CP000560.1|	**98.44**	**0.99765**	93.36	**97.84**	56.2 ± 2.74	46.5	**99.61**	Infected sugar beet
CC178	gb|CP006845.1|	**98.44**	**0.99764**	93.41	**97.84**	56.1 ± 2.74	46.5	**99.61**	Cucumber phyllosphere
AP183	JXAM01000000	**98.44**	**0.99725**	93.02	n.d.	55.3 ± 2.72	46.4	**99.67**	Cotton rhizosphere
KHG19	gb|CP007242.1|	**98.44**	**0.99757**	93.44	**97.82**	56.1 ± 2.74	46.6	**99.41**	Fermented soybean paste
UCMB5036	emb|HF563562.1|	**98.44**	**0.99727**	93.42	**97.83**	56.1 ± 2.74	46.6	**99.67**	Inner tissues of the cotton plant
HB-26	AUWK01000000	**98.44**	**0.99771**	93.28	**97.80**	55.5 ± 2.73	46.4	**99.61**	Soil from china
AH159-1	JFBZ01000000	**98.44**	**0.99815**	93.14	**96.68**	54.9 ± 2.72	46.4	**99.61**	Mushroom korea
AS43.3	gb|CP003838.1|	**98.41**	**0.99777**	93.51	**97.78**	55.9 ± 2.74	46.6	**99.67**	Surface of a wheat spike
UCMB5113	emb|HG328254.1|	**98.41**	**0.99732**	93.50	**97.80**	56.4 ± 2.75	46.7	**99.61**	Soil from karpaty mountains
IT-45	gb|CP004065.1|	**98.41**	**0.99755**	93.42	**97.18**	55.5 ± 2.73	46.6	**99.67**	Unknown
UASWS BA1	AWQY01000000	**98.41**	**0.99742**	93.49	**97.83**	55.4 ± 2.73	46.6	**99.61**	Inner wood tissues of platanus tree
GB03^*^	AYTJ00000000.1	**98.38**	**0.99715**	93.29	**97.78**	55.0 ± 2.72	46.6	n.d	Phyllosphere,douglas fir, australia
Pc3	gb|CP010406.1|	**98.38**	**0.99745**	93.38	**97.79**	56.0 ± 2.74	46.5	**99.67**	Antarctic seawater
TF28	ref|NZ_KN723307.1|	**98.38**	**0.99692**	93.26	**97.79**	55.5 ± 2.73	46.4	**99.76**	Soybean roots
G341	gb|CP011686.1|	**98.38**	**0.99793**	93.34	**97.78**	56.0 ± 2.74	46.5	**99.61**	Korean ginseng rhizosphere
EBL11	JCOC01000000	**98.38**	**0.99746**	93.44	**97.84**	55.9 ± 2.74	46.4	**99.61**	Rice rhizosphere
LPL-K103	JXAT01000000	**98.38**	**0.99713**	93.37	**97.80**	55.7 ± 2.73	46.6	**99.54**	Lemon slices
YJ11-1-4	gb|CP011347.1|	**98.38**	**0.99766**	93.07	**97.81**	55.5 ± 2.73	46.4	**99.67**	Korean doenjang soybean paste
ATCC 19217	gb|CP009749.1|	**98.38**	**0.99737**	93.08	**97.84**	55.6 ± 2.73	46.4	**99.67**	Industry (producer guanylic acid)
5B6	gb|AJST01000001.1|	**98.38**	**0.99774**	93.34	**97.79**	55.4 ± 2.73	46.6	**99.67**	Cherry tree phyllosphere
SQR9	gb|CP006890.1|	**98.35**	**0.99753**	93.08	**97.78**	55.6 ± 2.73	46.1	**99.67**	Cucumber rhizosphere
NJN-6	gb|CP007165.1|	**98.35**	**0.99823**	93.11	**97.84**	55.3 ± 2.72	46.6	**99.61**	Banana rhizosphere
LFB112	gb|CP006952.1|	**98.35**	**0.99772**	93.25	**97.84**	55.6 ± 2.73	46.7	**99.61**	Chinese herbs
JJ-D34	gb|CP011346.1|	**98.35**	**0.99779**	93.27	**97.79**	55.3 ± 2.73	46.2	**99.61**	Deonjang, fermented soybean paste
L-S60	gb|CP011278.1|	**98.32**	**0.99752**	93.44	**97.84**	55.3 ± 2.73	46.7	**99.61**	Turfy soil in beijing, china
L-H15	gb|CP010556.1|	**98.32**	**0.99753**	93.41	**97.84**	55.4 ± 2.73	46.7	**99.61**	Cucumber seedlings
M27	AMPK01000000	**98.32**	**0.99816**	93.32	**97.79**	55.5 ± 2.73	46.6	**99.61**	Cotton waste compost
B-1	gb|CP009684.1|	**98.30**	**0.99749**	93.37	**97.84**	55.2 ± 2.72	46.2	**99.48**	Oil field
Co1-6	emb|CVPA01000001	**98.30**	**0.99781**	93.34	**97.77**	55.4 ± 2.73	46.4	**99.67**	Calendula officinalis rhizosphere
KCTC13012^T^	LHCC00000000	**98.27**	**0.99752**	93.13	**97.78**	55.5 ± 2.73	46.4	n.d.	Mouth at the river velez, spain
B9601-Y2	emb|HE774679.1|	**98.27**	**0.99731**	93.16	**97.79**	55.9 ± 2.74	45.9	**99.81**	Wheat rhizosphere
BH072	gb|CP009938.1|	**98.27**	**0.99794**	93.32	**97.78**	56.0 ± 2.74	46.4	**99.81**	Honey sample
CAU B946	emb|HE617159.1|	**98.27**	**0.99796**	93.39	**97.80**	55.3 ± 2.73	46.5	**99.61**	Rice rhizosphere
NKYL29	JPYY01000000	**98.24**	**0.99719**	93.27	**97.79**	55.6 ± 2.73	46.3	n.d.	Ranzhuang tunnel, hebei, china
Lx-11	AUNG00000000.1	**98.21**	**0.99691**	93.28	**97.21**	55.0 ± 2.72	46.4	n.d.	Soil jiangsu province, china
KACC 13105^T^	AQGM00000000.1	**98.21**	**0.99685**	93.29	**97.75**	55.2 ± 2.72	46.4	**99.67**	Rice rhizosphere
X1	JQNZ01000000	**98.21**	**0.99741**	93.27	**97.75**	55.3 ± 2.73	46.5	**99.78**	Soil wuhan province, china
B-1895	JMEG01000000	**98.21**	**0.99816**	93.33	**97.82**	55.8 ± 2.73	46.2	**99.67**	Unknown
DC-12	AMQI01000000	**98.16**	**0.99785**	93.60	**97.84**	56.2 ± 2.74	46.1	**99.67**	Fermented soya beans
SK19.001	AOFO01000000	**98.13**	**0.99753**	93.55	**97.85**	56.5 ± 2.75	46.2	**99.77**	Unknown
***B. subtilis*** **subsp. subtilis**									
168	emb|AL009126.3|	90.26	0.95411	76.32	85.43	20.9 ± 2.33	43.5	**99.48**	Soil:several rounds of mutagenesis

Similarity (% identity) of the rpoB gene nucleotide sequence and of the 16S rRNA to DSM7^T^ is shown. AAI matrix median values against. DSM7^T^ and the G+C % content of the genomes are also presented. The Tetra correlation search (TCS) was performed with DSM7^T^ yielding 66 strains with ≥0.989 Z-score (boundary for species delineation). Formula 2 was used to estimate genome-to-genome distance comparisons (GGDC2.1) with the DSM7^T^ genome. Values exceeding species threshold are presented in bold letters. The type strains B. amyloliquefaciens DSM7^T^, B. siamensis KTCC 13613^T^, B. vanillea XY18^T^, B. amyloliquefaciens subsp. plantarum FZB42^T^, B. methylotrophicus KACC 13105^T^, and B. velezensis KCTC 13012^T^ are underlined. GB03^*^ and FZB42^*^ are strains used for commercial production of biofertilizers and biocontrol agents.

**Figure 2:**
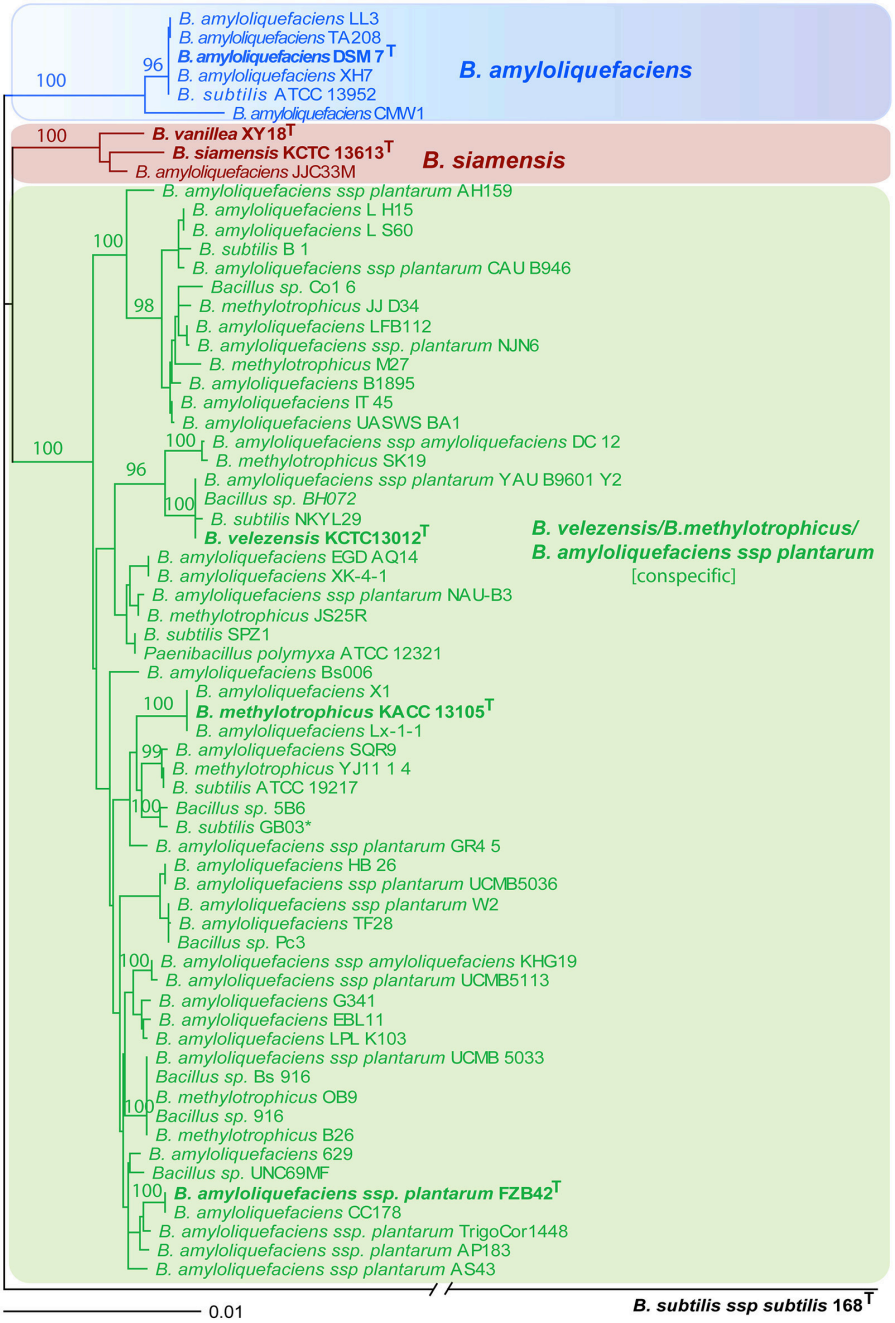
**NJ phylogenetic tree, extracted from 66 complete *rpoB* nucleotide sequences with high similarity to *B. amyloliquefaciens* DSM7^T^ (>98% identity)**. *B. subtilis* subsp. *Subtilis* 168^T^ was used as outgroup. The consensus tree was reconstructed from 1000 trees according to the extended majority rule (SEQBOOT program). Bootstrap values >90%, based on 1000 repetitions, are indicated at branch points. Strain and accession numbers are indicated. Type strains for *B. amyloliquefaciens* (DSM7^T^), *B. siamensis* (KCTC13613^T^) and *B. vanillea* (XY18^T^), and the conspecific group containing FZB42^T^ as the type strain for *B. amyloliquefaciens* subsp. *Plantarum, B. velezensis* KCTC13012^T^, and *B. methylotrophicus* KACC13105^T^ are in bold. Bar, 0.01 substitutions per nucleotide position. For further characterization of strains and genomes see Table 1.

The phylogenetic tree based on complete rpoB gene sequence suggests existence of three tightly connected monophyletic groups: (i) *B. amyloliquefaciens* containing six strains including type strain DSM7^T^; (ii) *B. siamensis* cluster consists of three strains: the type strain KCTC 13613^T^, strain XY18, originally assigned as type strain for *B. vanillea* (Chen et al., 2014) but recently reclassified as being *B. siamensis* (Dunlap, 2015), and a strain assigned as being *B. amyloliquefaciens* JJC33M; (3) the conspecific complex comprising *B. velezensis, B. methylotrophicus*, and *B. amyloliquefaciens* subsp. *Plantarum* contained 57 strains. The tree is robust displaying high bootstrap values for all three groupings, although the three clusters are closely related and separated by only 0.01–0.02 substitutions per nucleotide position. By contrast, taxonomic distance to *B. subtilis* is around tenfold larger (Figure 2).

### Phylogenomic analysis of clade II (operational group *B. amyloliquefaciens*)

**Figure 3:**
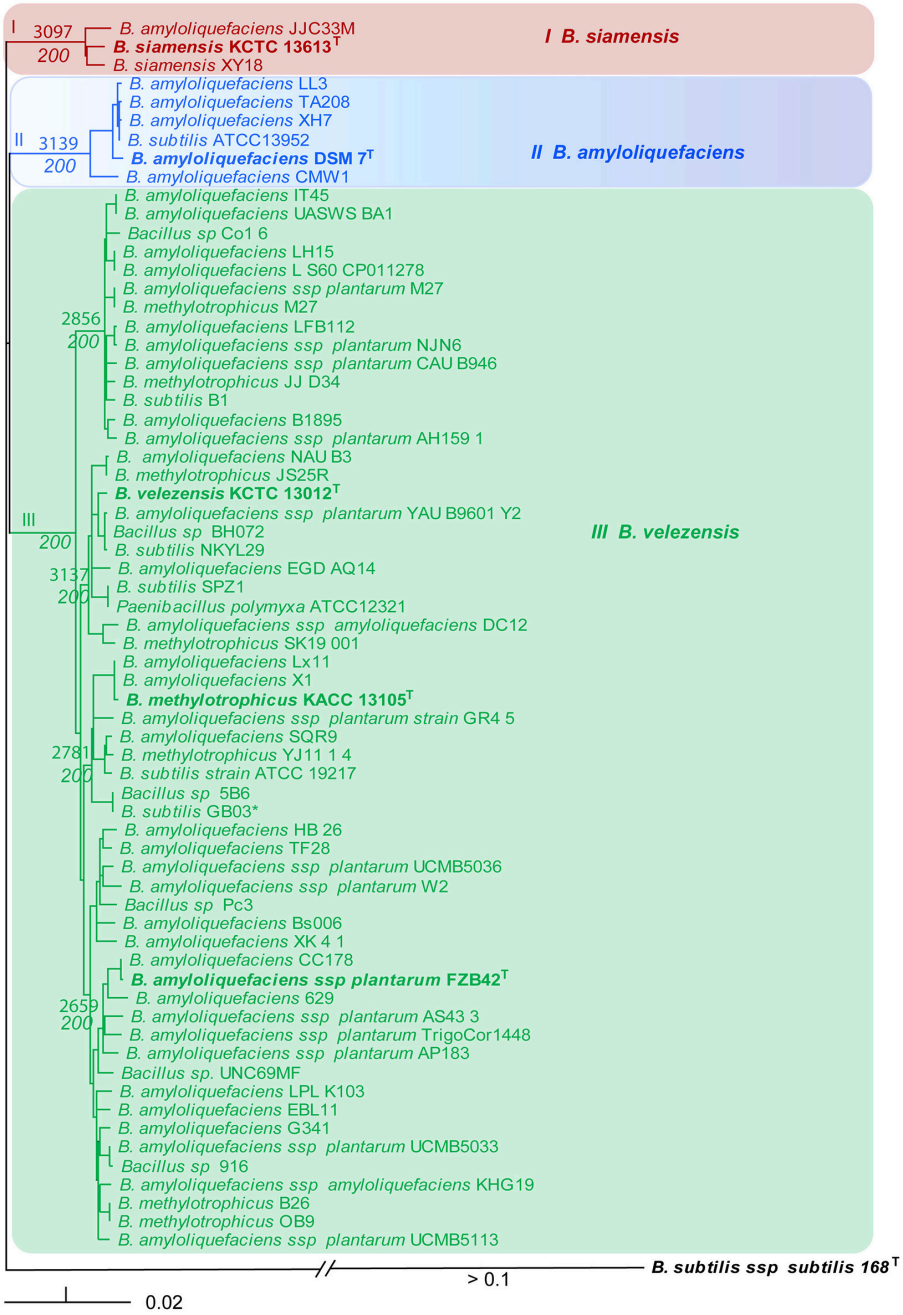
**NJ phylogenomic tree, constructed from the 66 core genomes with the highest similarity to DSM7^T^ (Table 1)**. The *B. subtilis* genome was used as outgroup. The number of core genome CDSs is indicated at the nodes. They were calculated for the respective subsets of genomes. Bootstrap values obtained from 200 repetitions are also indicated at the nodes. Type strains (T) are indicated by bold letters. Bar, 0.02 substitutions per nucleotide position.

In order to confirm the phylogenetic analysis based on *rpoB* sequences we calculated the core genomes using the EDGAR 1.3 program package. A total of 1998 CDSs were shared by the 66 core genome sequences extracted in that analysis. It ruled out that the phylogenomic tree based on complete core genome sequences (Figure 3) did reflect the phylogenomic distances similar as the phylogenetic tree based on *rpoB* nucleotide sequences (Figure 2). The same robust monophyletic groups as in Figure 2 were obtained. The *B. siamensis* cluster consisting of three representatives shared a core genome of 3097 CDSs; the *B. amyloliquefaciens* cluster consisting of six representatives shared a core genome of 3139 CDSs; and the conspecific group containing 57 plant-growth promoting Bacilli including FZB42^T^ shared a relatively small core genome consisting of only 2295 CDSs, which is mainly due to the high number of genomes included in this analysis. Subgroups of this cluster shared core genomes ranging from 2659 to 3137 CDSs (Figure 3). Again, the NJ tree suggested that *B. amyloliquefaciens* subsp. *Plantarum, B. methylotrophicus*, and *B. velezensis* formed a monophyletic group corroborating recent findings (Dunlap C. A. et al., 2015; Wu et al., 2015; Dunlap C. et al., 2016).

**Figure 4:**
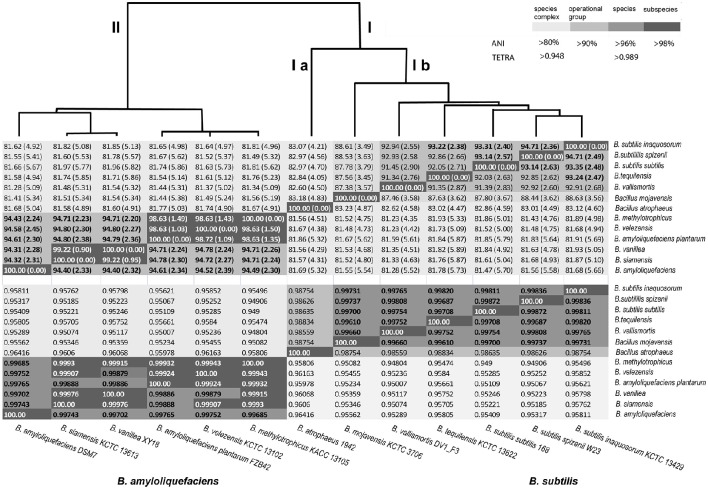
**Dendrogram of the type strains of clade I (operational group “*B.subtilis”*) and II (operational group “*B. amyloliquefaciens*”) based on their median ANIb values (upper part of the Table) and Tetra-nucleotide correlation signatures (lower part of the Table)**. The median nucleotide percent identity values between the orthologous genes of the core of the selected genomes after pairwise BLASTN comparison are indicated. Standard deviation values are given in parentheses.

At next we tried to elucidate the taxonomic status of these closely related genomes. Different phylogenetic and phylogenomic methods were used to analyze relationship of all 65 genomes with that of *B. amyloliquefaciens* DSM7^T^. As shown above, *rpoB* sequence similarity, exceeding threshold of species delineation, and the intraspecific Tetra-nucleotide signature correlation index (>0.99) suggested that all strains analyzed belong to the species *B. amyloliquefaciens*. TETRA analysis (Jspecies) demonstrated that the six type strains of clade II were closely related and yielded pairwise Tetra results (tetranucleotide signature correlation index) in species range (≥0.989, Figure 4 lower part). Deviations of the mean G+C content calculated for the whole genomes were less than one percent which does not contradict species definition (Table 2). Grouping of all strains into a single species, *B. amyloliquefaciens*, was further supported by the AAI values (Table 1). The mean AAI values of the 66 core genomes selected by their *rpoB* similarity to DSM7^T^ were ≥96.5%, exceeding the proposed cut-off of 96% for species delineation. However, parameters, considered recently as being most important for genome-based species delineation, such as ANI and dDDH (Federhen et al., 2016), did not support this conclusion (Table 1).

**Table 2:** **Summary of phylogenetic (*rpoB*) and phylogenomic parameters calculated for *B. amyloliquefaciens, B. siamensis* and conspecific group consisting of *B. amyloliquefaciens plantarum, B. methylotrophicus* and *B. velezensis* against corresponding type strains**.

** Reference/Query**	**G+C**	***rpoB***	**TETRA**	**ANIb**	**AAI**	**dDDH**
	%	(≥97%)	(≥0.989)	(≥96%)	(≥96%)	(≥70%; ≥79%)
***B. amyloliquefaciens*****/DSM7**^T^						
Mean	45.88	99.92	0.9994	99.19	99.63	94.52
Median	45.83	100	0.9994	99.30	99.91	95.40
*SD*	0.14	0.20	0.0004	0.74	0.22	5.14
*n*	6	6	6	6	6	6
***B. amyloliquefaciens*****/FZB42**^T^						
Mean	45.88	98.39	0.9980	93.79	96.63	55.90
Median	45.83	98.44	0.9979	93.75	97.43	55.70
*SD*	0.14	0.11	0.0003	0.11	0.12	0.13
*n*	6	6	6	6	6	6
***B. amyloliquefaciens*****/KCTC13613**						
Mean	45.88	98.27	0.9981	93.57	96.54	54.60
Median	45.83	98.27	0.9989	93.57	97.44	54.60
*SD*	0.14	0.01	0.0013	0.04	0.18	0.16
*n*	6	6	6	6	6	6
***B. siamensis/*****DSM7**^T^						
Mean	46.1	98.41	0.9970	93.27	96.48	54.67
Median	46.3	98.44	0.9950	93.27	97.41	54.70
*SD*	0.341	0.99	0.0013	0.009	0.08	0.35
*n*	3	3	3	3	3	3
***B. siamensis/*****FZB42**^T^						
Mean	46.1	98.67	0.9981	93.87	96.79	56.5
Median	46.3	98.65	0.9989	94.01	97.69	56.8
*SD*	0.341	0.10	0.0013	0.33	0.92	0.58
*n*	3	3	3	3	3	3
***B. siamensis/*****KCTC13613**						
Mean	46.1	99.70	0.9991	99.01	99.56	93.40
Median	46.3	99.64	0.9998	98.84	99.89	94.45
*SD*	0.341	0.27	0.001	0.92	0.39	5,86
*n*	3	3	3	3	3	3
**CONSPECIFIC GROUP** ***B. amyloliquefaciens plantarum, B. methylotrophicus, B. velezensis*****/DSM7^T^**						
Mean	46.39	98.39	0.9975	93.32	96.57	55.70
Median	46.45	98.40	0.9975	93.34	97.41	55.60
*SD*	0.245	0.09	0.0004	0.13	0.24	0.38
*n*	55	56	57	56	55	39
**CONSPECIFIC GROUP** ***B. amyloliquefaciens plantarum, B. methylotrophicus, B. velezensis*****/FZB42^T^**						
Mean	46.39	99.46	0.9991	98.30	99.11	87.10
Median	46.45	99.52	0.9994	98.28	99.49	86.50
*SD*	0.245	0.24	0.0004	0.575	0.39	4.89
*n*	55	56	57	56	55	31
**CONSPECIFIC GROUP** ***B. amyloliquefaciens plantarum, B. methylotrophicus, B. velezensis*****/ KCTC13613**						
Mean	46.39	98.46	0.9987	94.11	96.97	56.90
Median	46.45	98.50	0.9989	94.12	97.80	56.85
*SD*	0.245	0.109	0.0013	0.079	0.23	0.15
*n*	55	57	57	56	55	33

*Threshold values for species and, in case of dDDH, subspecies delineation are given in parentheses. SD, standard deviation; n, number of samples. Values indicating one species are presented in green fields*.

ANI analysis performed with the EDGAR program package discriminated clearly two clusters corresponding to clades I and II of the *B. subtilis* species complex (Figure 4). Clade II representing the *B. amyloliquefaciens* group was divided into three groups consisting of *B. amyloliquefaciens* DSM7^T^ (i), *B. siamensis* and *B. vanillea* (ii), and the conspecific complex formed by the type strains *B. methylotrophicus* KACC13105^T^, *B. velezensis* KCTC 13102^T^ and *B. amyloliquefaciens* subsp. *plantarum* FZB42^T^ (iii). The latter group displayed ANI values of >98% exceeding the cut-off for species delineation when compared with each other suggesting that the members of the conspecific complex belong to a single species. *B. methylotrophicus* KACC 13105^T^, *B. velezensis* KCTC 13102^T^, and *B. amyloliquefaciens* subsp. *plantarum* FZB42^T^ displayed similar median ANI values ranging between 94.3 and 94.8% when compared with *B. amyloliquefaciens* DSM7^T^ (1) and *B. siamensis* KCTC-13613^T^ (2), respectively. Given a calculated deviation of ±2.2–2.3% the ANI matrix values suggests a high degree of relatedness to *B. amyloliquefaciens, B. siamensis* and the conspecific group formed by *B. amyloliquefaciens* subsp. *plantarum, B. methylotrophicus*, and *B. velezensis*, but did not sufficiently support species delineation (Figure 4, upper part). According to more recent findings the recommended cut-off point for species delineation corresponds to ~96% ANI (Colston et al., 2014). Similar results were obtained when ANIb and ANIm values were determined by using the JSpecies program package for all the 66 genomes included in this analysis. Threshold values sufficient for species delineation were only obtained, when representatives of *B. amyloliquefaciens* (6 genomes), *B. siamensis* (3 genomes), and of the conspecific group (57 genomes) were compared with their respective type strains. However, comparison of the 57 strains of the conspecific group (e.g., FZB42, *B. velezensis*) with either *B. amyloliquefaciens* DSM7^T^ or *B. siamensis* KCTC 13613^T^ yielded ANI values slightly below the cut-off for species delineation. The same was true when the three members of the *B. siamensis* group were compared with either FZB42^T^ or DSM7^T^ (Table 2) suggesting that according to ANI analysis the members of clade II represent three discrete, although closely related, species.

In order to finally decide, whether all strains of clade II belong to one species or not, electronic DNA-DNA hybridization (dDDH) was applied in a quantitative analysis involving all 66 genomes. As shown previously, dDDH is useful to mimic the wet-lab DDH and can be used for genome-based species delineation and genome-based subspecies delineation (Meier-Kolthoff et al., 2013, 2014). For calculating dDDH three different formulas can be applied (see Materials and Methods), but only results obtained with the recommended formula 2 were used in our analysis (Table 2). When comparing members of the “*siamensis* group 2” and the “conspecific *B. velezensis* group” with *B. amyloliquefaciens* DSM7^T^, dDDH values of <70%, the defined threshold for species delineation, were obtained. All in all, dDDH supports our previous finding about a close relationship within clade II, but did not support their classification into one single species. The results are summarized in Table 2 and Supplementary Table 1.

### Gene clusters involved in nonribosomal synthesis of secondary metabolites

Compared to other members of the *B. subtilis* species complex, the plant-associated *B. amyloliquefaciens* possess an enormous potential to synthesize bioactive secondary metabolites. Besides five gene clusters, known from *B. subtilis* to mediate nonribosomal synthesis of secondary metabolites, four giant gene clusters absent in *B. subtilis* 168 were identified in FZB42 (Chen et al., 2007). The nine gene clusters that direct the synthesis of bioactive peptides and polyketides by modularly organized mega-enzymes define both nonribosomal peptide synthetases (NRPSs) and polyketide synthases (PKS). Three (*bmyD, dfn*, and *mln*) are not present in *B. subtilis* 168, but occur in all members of the “operational group *B. amyloliquefaciens.”* Except for the gene cluster encoding bacilysin synthesis, the functional activities of the remaining gene clusters depend on Sfp, an enzyme that transfers 4′-phosphopantetheine from coenzyme A to the carrier proteins of nascent peptide or polyketide chains. A direct comparison revealed that the nine gene cluster responsible for nonribosomal synthesis of bioactive secondary metabolites including macrolactin are only present in FZB42 and in the other members of the conspecific *B. velezensis* group, whilst the gene cluster involved in macrolactin synthesis was not detected in *B. siamensis* and *B. amyloliquefaciens* (Table 3). Noteworthy, the gene cluster responsible for synthesis of the polyketide difficidin was present in *B. siamensis*, but not in any other member of the *B. subtilis* species complex suggesting a stepwise loss of the ability to synthesize secondary metabolites in the order *B. velezensis* (including FZB42) 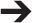 *B. siamensis* 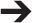 *B. amyloliquefaciens*.

**Table 3:** **Gene clusters encoding nonribosomal synthesis of lipopeptides and polyketides in type strains of *B. subtilis, B. amyloliquefaciens, B. siamensis*, and *B. velezensis***.

**Lipopeptides**	**FZB42**		***B. subtilis***		***B. amyloliquefaciens***		***B. siamensis***		***B. velezensis***	
**Surfactin**	BGC0000433									
Genes	Accession	bp	Accession	bp	Accession	bp	Accession	bp	Accession	bp
srfAA	RBAM_003650	10755	ssp168_402	10764	bAMF_0312	10755	RS09245	10755		
srfAB	RBAM_003660	10761	ssp168_403	10752	bAMF_0313	10761	RS09240	10761		
srfAC	RBAM_003680	3837	ssp168_405	3828	bAMF 0314	3814	RS09235	3837	AKJ10_17500	3837
srfAD	RBAM_003690	732	ssp168_406	729	bAMF 0315	732	RS09230	732	AKJ10_17505	732
tpaat	RBAM_003700	1311	ssp168_407		bAMF_0316	1311	RS09225	1311	AKJ10_17510	1311
**BacillomycinD**	BGC0001090									
xynD	RBAM_018150	1539	ssp168_1991	1539	bAMF_1910	1536	RS05225	1539	AKJ10_09355	1539
bmyC	RBAM_018160	7860			bAMF_1911	7851	RS05230	7857	AKJ10_09360	7860
bmyB	RBAM_018170	16092			bAMF_1912	16086	RS05235	16083	AKJ10_09365	16092
bmyA	RBAM_018180	11949			bAMF_1913	11949	RS05240	10137	AKJ10_09370	11949
bmyD	RBAM_018190	1203			bAMF_1914	1242	RS16690	1203	AKJ10_09375	1203
yxjF	RBAM_018200	786	ssp168_4194	786	bAMF_1916	786	RS16685	786	AKJ10_09380	
**Fengycin**	BGC0001095									
yngL	RBAM_018410	432	ssp168_2005	393	bAMF_1937	381	RS16575	381	AKJ10_09485	381
fenE	RBAM_018420	3804	ssp168_2006	3840	bAMF_1938	3807	RS16570	3804	AKJ10_09490	3804
fenD	RBAM_018430	10776	ssp168_2007	10812	bAMF_1939	7677	RS16565	10776	AKJ10_09495	4431
fenC	RBAM_018440	7650	ssp168_2008	7668			RS16560	4584	AKJ10_19615	4395
fenB	RBAM_018450	7698	ssp168_2009	7683			RS15850	7704	AKJ10_19590	7698
fenA	RBAM_018460	7659	ssp168_2010	7686			RS15845	4605		
dacC	RBAM_018470	1476	ssp168_2011	1476	bAMF_1940	1476	RS08800	1476	AKJ10_19155	1476
**POLYKETIDES**										
**Macrolactin**	BGC0000181_c1									
ykyA	RBAM_014310	639	ssp168_1616	672	bAMF_1532	663	RS0102445	663	AKJ10_06145	639
	RBAM_014320	168							AKJ10_06140	210
mlnA	RBAM_014330	2307							AKJ10_06135	2307
mlnB	RBAM_014340	12261							AKJ10_06130	12258
mlnC	RBAM_014350	4773							AKJ10_06125	4773
mlnD	RBAM_014360	8709							AKJ10_06120	8712
mlnE	RBAM_014370	7005							AKJ10_06115	7005
mlnF	RBAM_014380	5712							AKJ10_06110	5712
mlnG	RBAM_014390	7383							AKJ10_06105	7383
mlnH	RBAM_014400	3852							AKJ10_06100	3849
mlnI	RBAM_014410	1092							AKJ10_06095	1092
pdhA	RBAM_014420		ssp168_1617	1116	bAMF_1533	1116	RS15370	1116	AKJ10_06090	1116
**Bacillaene**	BGC0001089_c									
mutL	RBAM_016890	1875	ssp168_1874	1884	bAMF_1777	1884	RS0101170	1878	AKJ10_04875	1875
baeB	RBAM_016900	678	ssp168_1878	678	bAMF_1778	678	RS0101150	678	AKJ10_04835	678
baeC	RBAM_016910	870	ssp168_1879	867	bAMF_1779	870	RS0101145	870	AKJ10_04830	870
baeD	RBAM_016920	975	ssp168_1880	975	bAMF_1780	975	RS0101140	975	AKJ10_04825	975
baeE	RBAM_016930	2241	ssp168_1881	2304	bAMF_1781	2241	RS0101135	2241	AKJ10_04820	2241
acpK	RBAM_016940	249	ssp168_1882	249	bAMF_1782	249	RS0101130	249	AKJ10_04815	249
baeG	RBAM_016950	1263	ssp168_1884	1263	bAMF_1783	1263	RS0101125	1263	AKJ10_04810	1263
baeH	RBAM_016960	774	ssp168_1885	780	bAMF_1784	774	RS0101120	774	AKJ10_04805	774
baeI	RBAM_016970	750	ssp168_1886	750	bAMF_1785	750	RS0101115	750	AKJ10_04800	750
baeR	RBAM_017020	7449	ssp168_1891	7632	bAMF_1790	7446	RS0101090	7455	AKJ10_04775	7458
baeS	RBAM_017030	1212	ssp168_1892	1218	bAMF_1791	1212	RS0101085	1212	AKJ10_04770	1212
baeJ	RBAM_016980	14949	ssp168_1887	15132	bAMF_1786	14952	RS0101110	14931	AKJ10_04795	14946
baeL	RBAM_016990	13428	ssp168_1888	13617	bAMF_1787	13431	RS0101105	13392	AKJ10_04790	13413
baeM	RBAM_017000	10536	ssp168_1889	12789	bAMF_1788	10542	RS0101100	10506	AKJ10_04785	10536
baeN	RBAM_017010	16302	ssp168_1890	16467	bAMF_1789	16314	RS0101095	16293	AKJ10_04780	16305
**Difficidin**	BGC0000176_c1									
proI	RBAM_021930	840	ssp168_2591	837	bAMF_2277	843	RS0119580	843	AKJ10_01435	840
dfnM	RBAM_021940	747					RS0119585	747	AKJ10_01440	747
dfnL	RBAM_021950	1248					RS0119590	1248	AKJ10_01445	1248
dfnK	RBAM_021960	1155					RS0119595	1155	AKJ10_01450	1155
dfnJ	RBAM_021970	6216					RS0119600	6216	AKJ10_01455	6216
dfnI	RBAM_021980	6153					RS0119605	6156	AKJ10_01460	6156
dfnH	RBAM_021990	7719					RS0119610	7719	AKJ10_01465	7719
dfnG	RBAM_022000	15615					RS0119725	8904	AKJ10_01470	15615
dfnF	RBAM_022010	5727					RS0119720	5727	AKJ10_01475	5727
dfnE	RBAM_022020	6297					RS0119715	6285	AKJ10_01480	6297
dfnD	RBAM_022030	12591					RS0119615	15654	AKJ10_01485	9252
dfnC	RBAM_022040	738					RS0119620	738	AKJ10_01490	738
dfnB	RBAM_022050	1332					RS0119625	1371	AKJ10_01495	1365
dfnX	RBAM_022060	273					RS0119630	273	AKJ10_01500	273
dfnY	RBAM_022070	981					RS0119635	981	AKJ10_01505	981
dfnA	RBAM_022080	2259					RS0119640	2259	AKJ10_01510	2259

*The genes occurring in plant-growth-promoting FZB42 were used for reference. The MIBiG specifications (Medema et al., 2015) of the FZB42 gene clusters involved in synthesis of secondary metabolites are indicated*.

## Discussion

The *B. subtilis* species complex consists of a steadily increasing number of validly described species (see Introduction), which display an extremely high degree of similarity. They are very difficult to distinguish by using classical taxonomy parameters: morphological and physiological characteristics, cell wall compositions, 16S rRNA sequence, G+C content, and FAME. Also, experimental determination of DNA-DNA relatedness (DDH), gold-standard of bacterial taxonomy for 50 years, yields often erroneous and variable results (Auch et al., 2010). Therefore, the taxonomic status of these species constantly brings confusion to researchers, especially for non-professional taxonomy researchers. Our analysis using the available core genomes of 23 type strains suggests that within the *B. subtilis* species complex four clades can be distinguished: clade I consisting of *B. subtilis* including its three subspecies *subtilis, spizenii*, and *inaquosorum, B. tequilensis, B. vallismortis, B. mojavensis*, and *B. atrophaeus*, clade II consisting of *B. siamensis, B. amyloliquefaciens*, and a conspecific complex consisting of *B. methylotrophicus, B. velezensis*, and *B. amyloliquefaciens* subsp. p*lantarum*, clade III consisting of *B. licheniformis* and related species, and clade IV consisting of *B. pumilus* and related species (Figure 1).

We have chosen here clade II comprising *B. amyloliquefaciens* and related species for a deeper analysis. Due to the high number of available genomic sequences, we applied a quantitative phylogenomic approach including 66 genomes with a high degree of similarity to DSM7^T^, the type strain of *B. amyloliquefaciens*. In accordance to Dunlap C. A. et al. (2015) we could demonstrate existence of three distinct monophyletic groups within this clade. Six core genomes represented the species *B. amyloliquefaciens* and three strains were assigned as being *B. siamensis*. The results of our extensive phylogenomic analysis (Table 2) corroborates the monophyletic nature of the conspecific group consisting of *B. amyloliquefaciens* subsp. *plantarum, B. methylotrophicus*, and *B. velezensis*, suggesting that this unique taxon is closely related to *B. amyloliquefaciens* (Borriss et al., 2011). *B. velezensis* is a heterotypic synonym of *B. methylotrophicus, B. amyloliquefaciens* subsp. *plantarum*, and *Bacillus oryzicola*, and is used to control plant fungal diseases. This idea is further supported by a recent phylogenetic and phylogenomic analysis in which *B. amyloliquefaciens, B. siamensis*, and *B. amyloliquefaciens* subsp. *plantarum* were established as closely related monophyletic groups harboring a common ancestor based on their *gyrB* and core genome (729,383 bp) sequences (Hossain et al., 2015). The conspecific group consisting of *B. amyloliquefaciens* subsp. *plantarum, B. methylotrophicus*, and *B. velezensis* was recently classified as being *B. velezensis* (Dunlap C. et al., 2016), because the valid publication of *B. velezensis* (Ruiz-García et al., 2005a) predates the publication of *B. methylotrophicus* (Madhaiyan et al., 2010) and *B. amyloliquefaciens* subsp. *plantarum* (Borriss et al., 2011). The tight relatedness of *B. siamensis* and *B. velezensis* with *B. amyloliquefaciens* is indicated by:

- highly conserved *rpoB* nucleotide sequence with more than 98% identity to DSM7^T^;
- Mean G+C % content is only 0.5% different ranging between 45.9% (subsp. *amyloliquefaciens*), 46.1% (subsp. *siamensis*), and 46.4% (subsp. *plantarum*);
- Tetranucleotide signatures, TETRA, were determined as above the cut-off for species delineation (>0.989);
- AAI values are well above 96%, representing the intraspecific threshold.

On the other hand, ANIb and ANIm were calculated as around 93 to 94% identity to *B. amyloliquefaciens* on the nucleotide level, which is slightly lower than the threshold proposed for species delineation (95–96% ANI, Kim et al., 2014). Moreover, electronic DDH calculation using formula 2 yielded only 56% identity, which is clearly below the cut-off for species delineation. In spite of these contradictory results, we have to conclude that three discrete species exist within clade II, given that results obtained by ANI and dDDH are more important in modern taxonomy (Auch et al., 2010; Meier-Kolthoff et al., 2013) and outcompete the other results favoring a *B. amyloliquefaciens* subspecies concept. However, due to the close relationship of all three species comprised in clade II we propose an “operational group *B. amyloliquefaciens*” comprising *B. amyloliquefaciens, B. siamensis*, and *B. velezensis*. The introduction of this “operational group” above species level should improve hierarchical classification within the *B. subtilis* species complex. The members of the “operational group *B. amyloliquefaciens* are distinguished from *B. subtilis* and its closest relatives by their ability to synthesize nonribosomally antifungal acting lipopopeptides of the iturin group, mostly bacillomycin D or iturin A. The ecotype of plant-associated *B. amyloliquefaciens* is well introduced since many years (Reva et al., 2004) and includes the most important biocontrol- and plant-growth-promoting Bacilli, which are successfully used as environmental-friendly means in agriculture (Borriss, 2011). In addition, numerous studies have been published in recent years in order to identify and to understand the specific features of the group of *B. amyloliquefaciens* strains able to colonize plant organs and to withstand strong plant response reactions. As in *B. subtilis* it is now widely recognized that a relevant part of metabolism of plant-associated *B. amyloliquefaciens* is devoted to metabolic interactions with plants (Belda et al., 2013). Metabolites produced by the plant-associated *B. amyloliquefaciens* FZB42 and other members of the conspecific *B. velezensis* group represent a substantial part of the diversity of nonribosomal secondary metabolites from the genus Bacillus. For example, they produce three types of polyene polyketides (difficidin, macrolactin, and bacillaene) with strong antibiotic action (Chen et al., 2007). By contrast, *B. siamensis* does only produce two (difficidin and bacillaene) and soil-borne *B. amyloliquefaciens* does only produce one polyketide (bacillaene). It is highly desirable to apply a correct taxonomic designation to distinguish the plant-associated (= *B. velezensis*) and the soil-borne *B. amyloliquefaciens”* (= *B. amyloliquefaciens*) strains, but also to take into consideration their high degree of relatedness. This should be reflected by their grouping into the “operational *B. amyloliquefaciens* group,” as a novel taxonomic unit above species level but below the “*B. subtilis* species complex.” Introduction of the novel taxonomic unit seems also be recommended in spite of a permanent misuse in describing taxonomy important *Bacillus* biocontrol strains such as GB03 (Choi et al., 2014) and QST713 (Kinsella et al., 2009), which are often designated as *B. subtilis* although they are true representatives of *B. velezensis* and simultaneously members of the “operational group *B. amyloliquefaciens.”*

In summary, due to their differences in ANI and dDDH values, which are slightly below species level thresholds, we propose that *B. amyloliquefaciens, B. velezensis*, and *B. siamensis* should keep their status as species of its own, as proposed by Dunlap (Dunlap C. et al., 2016). The close relatedness of the three species is well reflected by the novel taxonomic unit “operational group *B. amyloliquefaciens*.” Introducing of this novel taxonomic unit should improve also understanding of previous and recent scientific investigations performed with “plant-associated *B. amyloliquefaciens*” strains which often have not been designated correctly.

Another less surprising finding from our analysis was that many of the publically available *Bacillus* genomes that we analyzed are inconsistently assigned. Fortunately, a recent initiative has been started to correct such mistakes in Genbank entries (Federhen et al., 2016).

## Author contributions

BF, JB, HK, and RB performed phylogenomic analyses. All authors were involved in preparing the manuscript. The final version of the manuscript was prepared by RB.

## Funding

The financial support for BF by the National Natural Science Foundation of China (No. 31100081), the Priority Academic Program Development (PAPD) of Jiangsu Higher Education Institutions, and Natural Science Foundation of Jiangsu Province (No. BK20151514) is gratefully acknowledged.

### Conflict of interest statement

The authors declare that the research was conducted in the absence of any commercial or financial relationships that could be construed as a potential conflict of interest.

## Abbreviations

AAI: average amino acid identity
ANI: average nucleotide identity
CDS: coding sequence
DDH: DNA–DNA hybridization
dDDH: digital DNA–DNA hybridization
GGDC: Genome-to-Genome Distance Calculator
TETRA: tetranucleotide frequency distribution.
